# Combined PLIBEL and RULA assessment of ergonomic risks across 12 operating room nursing procedures: a cross-sectional observational study

**DOI:** 10.1038/s41598-026-67002-1

**Published:** 2026-08-18

**Authors:** Yichao Yao, Qi Zhang, Yijing Ma

**Affiliations:** 1 Department of Operating Room, Baoding First Central Hospital, Baoding, China; 2 Department of Gastroenterology, Baoding First Central Hospital, Baoding, China

**Keywords:** Operating room nursing, Work-related musculoskeletal disorders, Ergonomic risk assessment, PLIBEL, RULA, Health care, Health occupations, Medical research, Risk factors

## Abstract

Operating room (OR) nurses face elevated risks of work-related musculoskeletal disorders (WMSDs) due to sustained awkward postures, heavy patient handling, and prolonged static loading. However, procedure-level ergonomic risk profiles across the full spectrum of OR nursing tasks remain inadequately characterized. A cross-sectional observational study was conducted among 25 OR nurses in a tertiary hospital in Baoding, China. Twelve common nursing procedures were systematically evaluated using the Swedish Ergonomic Hazard Identification Method (PLIBEL) for qualitative hazard screening and the Rapid Upper Limb Assessment (RULA) for quantitative risk scoring. Joint angles were measured using Kinovea (version 2025.1.0) motion analysis software to ensure objective RULA scoring. Inter-rater reliability was maintained through dual-assessor evaluation and consensus. PLIBEL identified a total of 243 adverse ergonomic factors across the 12 procedures, with individual procedures ranging from 13 to 25 factors. All procedures shared two common exposures: unsupported standing posture and fatiguing leg work. RULA grand scores ranged from 4 to 7, corresponding to risk levels II through IV. Five procedures achieved the maximum score of 7 (Level IV, very high risk): lateral positioning, prone positioning, postoperative patient transfer, stretcher transport, and equipment transport. These high-risk procedures were characterized by severe trunk flexion, asymmetric leg loading, and external loads exceeding 10 kg. Six procedures scored in the medium-risk range (RULA 5–6), while needle threading scored 4 (Level II, low risk). PLIBEL and RULA findings converged to identify patient positioning and transfer tasks as the highest-priority targets for ergonomic intervention. Adverse ergonomic factors are prevalent across common OR nursing procedures, with patient positioning and transfer tasks posing the most prominent risks. The combined application of PLIBEL and RULA offers complementary strengths—PLIBEL enabling comprehensive hazard identification and RULA providing graded risk quantification—generating actionable, procedure-specific risk profiles that identify the highest-priority targets for ergonomic intervention.

## Introduction

Work-related musculoskeletal disorders (WMSDs) represent one of the most prevalent occupational health problems affecting healthcare professionals worldwide. These disorders, characterized by pain, discomfort, and functional impairment in muscles, tendons, joints, and nerves, result primarily from sustained awkward postures, repetitive motions, forceful exertions, and prolonged static loading in the workplace^1^. Among all healthcare workers, nurses consistently report the highest rates of WMSDs, with operating room (OR) nurses facing particularly elevated risks due to the distinctive physical demands of surgical environments^2^. A recent systematic review and meta-analysis encompassing 22 international studies and 3,590 perioperative nurses reported pooled WMSDs prevalences of 62% for the lower back, 47% for the knees, and 44% for the shoulders^3^. These findings underscore that OR nurses are routinely exposed to heavy lifting, prolonged standing, forced postures, and repetitive instrument handling over extended surgical procedures.

The ergonomic burden on OR nurses is further amplified by the nature of their routine tasks, which frequently involve patient positioning, intraoperative instrument transfer, equipment transport, and postoperative patient transfer. Tavakkol et al. demonstrated that nursing staff in operating rooms exhibited the highest Rapid Entire Body Assessment (REBA) score (10/15) and Rapid Upper Limb Assessment (RULA) score (7/7) among all hospital departments, with 98% of OR nurses reporting musculoskeletal pain in the preceding 12 months^4^. Similarly, Ocaktan and Karabacak confirmed that OR nurses frequently adopt medium- to high-risk postures during surgical procedures, with the neck and lower back being the most severely affected body regions, and that REBA scores were significantly correlated with musculoskeletal symptoms^5^. These converging lines of evidence highlight an urgent need for systematic ergonomic risk identification and quantitative assessment specifically tailored to OR nursing tasks.

Several standardized observational tools have been developed for ergonomic risk assessment in occupational settings. Among these, the Swedish Ergonomic Hazard Identification Method (PLIBEL) provides a qualitative, checklist-based approach for systematically identifying adverse ergonomic factors across five body regions (neck, shoulder, and upper back; elbow, forearm, and hand; foot; knee and hip; and lower back), encompassing 17 hazard categories related to posture, load, tool use, and environmental constraints^6^. In parallel, the Rapid Upper Limb Assessment (RULA) method offers a quantitative scoring framework that evaluates postural deviations, muscle use, and external load to derive a composite risk score ranging from 1 (negligible risk) to 7 (very high risk), with corresponding action levels (Levels I–IV)^7^. While each tool has been individually validated across various occupational contexts, their combined application has been proposed to achieve a comprehensive assessment paradigm—with PLIBEL enabling broad-spectrum hazard screening and RULA providing graded risk quantification.

Previous studies have demonstrated the utility of PLIBEL and RULA as complementary tools in specific healthcare settings. Shen et al. and Wang et al. each applied this combined approach to evaluate ergonomic hazards in intravenous drug preparation tasks and gastrointestinal endoscopy nursing procedures, respectively, confirming that PLIBEL identified multiple adverse factors concentrated in the neck–shoulder–upper-back region, while RULA scores indicated medium-to-high risk levels^8,9^. A growing body of literature has also applied RULA independently to assess ergonomic risks among OR staff, consistently demonstrating elevated postural risk scores during surgical procedures^10,11^. However, despite these important contributions, no study to date has systematically applied PLIBEL and RULA in combination to assess the ergonomic risks inherent in the full spectrum of common OR nursing procedures—from preoperative preparation to intraoperative cooperation and postoperative finishing. This gap is particularly noteworthy given that OR tasks differ substantially from those in other clinical settings in terms of load magnitude, postural constraints, and task complexity.

Therefore, the present study aimed to employ both PLIBEL and RULA to conduct a systematic identification and quantitative assessment of adverse ergonomic factors across 12 common OR nursing procedures, including surgical hand antisepsis, sterile instrument table preparation, patient positioning (lateral and prone), surgical draping, needle threading, instrument transfer, postoperative instrument counting and sorting, postoperative patient transfer, dressing transport, stretcher patient transport, and equipment transport. By clarifying the high-risk procedures and their corresponding risk grades, this study sought to provide an evidence-based foundation for developing targeted ergonomic interventions aimed at preventing WMSDs among OR nurses.

## Methods

### Study design and participants

A cross-sectional observational study was conducted in the operating room of a tertiary first-class hospital in Baoding, Hebei Province, China. Operating room nurses were recruited according to predefined eligibility criteria. The recruitment period spanned from October 1, 2025, to October 31, 2025. Inclusion criteria were: (1) possession of a valid nursing license; (2) at least one year of operating room experience with proficiency in routine OR nursing procedures; and (3) voluntary participation with informed consent. Exclusion criteria were: (1) musculoskeletal injuries attributable to non-occupational causes (e.g., trauma, congenital conditions, pregnancy); (2) nursing interns or trainees; and (3) nurses who were pregnant or breastfeeding.

All participants provided written informed consent prior to enrollment. The consent form, approved by the ethics committee, outlined the study purpose, procedures, potential risks and benefits, confidentiality protections, and the voluntary nature of participation with the right to withdraw at any time without penalty. The consent forms were signed by each participant and retained by the research team. This study was conducted in accordance with the principles of the Declaration of Helsinki. The study protocol was approved by the Medical Ethics Committee of Baoding First Central Hospital (Approval No. [2025]015).

The minimum sample size was estimated based on precision considerations for estimating the intraclass correlation coefficient (ICC) in reliability studies. According to Gwet (2014), the approximate number of subjects required to obtain a confidence interval of desired width for an ICC can be determined using the following formula:

$$\:n=\frac{8\times\:{z}_{\alpha\:/2}^{2}\times\:{（1-{\uprho\:}）}^{2}}{{L}^{2}}＋1$$

where *ρ* is the anticipated ICC, *z*_*α*/2_ is the critical value of the standard normal distribution for a 95% confidence level ( *z*_*0.025*_=1.96 ), and *L* is the desired total width of the confidence interval.

Assuming an anticipated ICC of 0.80 for inter-rater reliability of RULA scoring, a desired 95% confidence interval total width (*L*) of 0.30, and α = 0.05 the estimated minimum sample size was approximately 20 participants. To account for potential variability in video-recorded procedures, we conservatively enrolled 25 nurses per procedure. This sample size is consistent with Gwet‘s^12^ recommendation that 20–30 subjects are typically sufficient when the anticipated ICC is ≥ 0.80-The minimum sample size was estimated at 20–25 nurses per procedure, based on an anticipated intraclass correlation coefficient (ICC) of 0.80 for inter-rater reliability of RULA scoring, a margin of error of 0.10, and a 95% confidence level, in accordance with sample size recommendations for agreement studies^4^. A total of 25 OR nurses were enrolled and observed for each of the 12 procedures.

## Selection of nursing procedures and key posture moments

Through systematic consultation with three senior OR nurses (each with ≥ 10 years of OR experience), 12 common OR nursing procedures were selected for ergonomic assessment. For each procedure, a single representative posture frame was identified as the key posture moment for both PLIBEL and RULA evaluation. This frame was defined as the moment with the greatest physical load, the largest postural deviation from the neutral position, and the highest representativeness. The selection of the key posture moment for each procedure was performed through discussion and consensus among three senior OR nurses (each with ≥ 10 years of clinical experience) and two research assessors trained in ergonomic assessment methods. The selection criteria were predefined as: (1) the moment of maximal physical exertion or external load; (2) the moment of greatest postural deviation from the anatomical neutral position across all body segments; and (3) the moment judged by the senior nurses to be most representative of the procedure’s routine execution. The two research assessors independently reviewed video recordings of the complete procedures to propose candidate key posture frames. The final frame for each procedure was determined through group discussion involving both the senior nurses and the assessors, with disagreements resolved by consensus. The operational definitions of the key posture moments for all 12 procedures are detailed in Table 1.

## PLIBEL and RULA assessment procedure

PLIBEL assessment was conducted independently by two trained assessors who evaluated each procedure against the 17-item checklist covering five body regions^6^. Disagreements between assessors were resolved through discussion. An adverse factor was considered present when observed in more than 80% of observations.

**Fig. 1:**
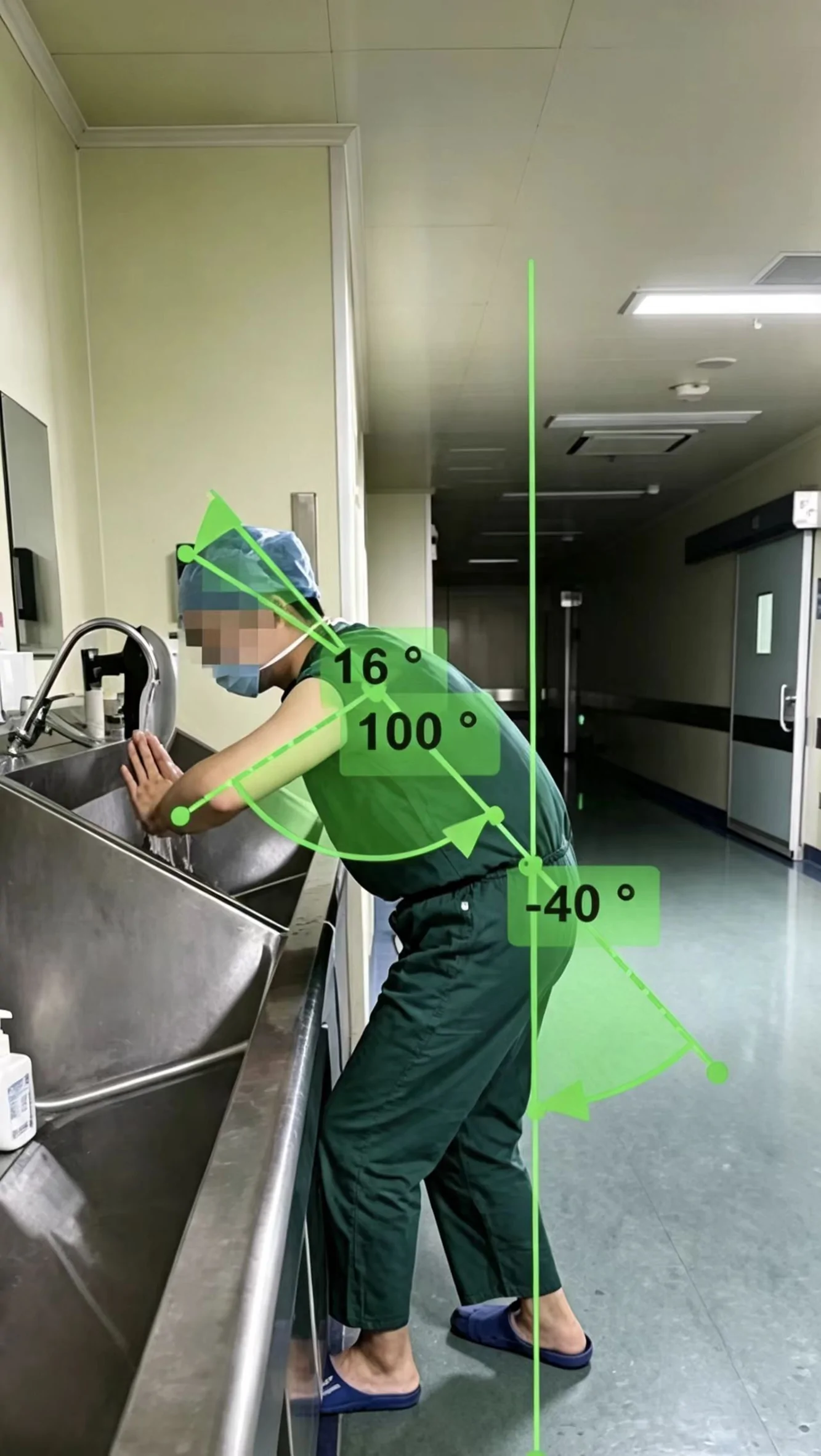
Representative frame of joint angle measurement using Kinovea software (surgical hand antisepsis) The figure shows a lateral view of the key posture moment with annotated joint angles: trunk flexion (40° relative to the vertical line), upper arm elevation (100° relative to the trunk), and neck flexion (16° relative to the trunk).These angle measurements were used to derive RULA posture scores for this procedure.

RULA assessment was performed by converting the Kinovea-measured joint angles into posture scores according to the standardized RULA scoring protocol^7^. Posture scores for the A-group (upper arm, forearm, wrist, wrist twist) and B-group (neck, trunk, legs) were cross-referenced with RULA Tables A and B to obtain the A- and B-group posture scores, respectively. Muscle use scores (+ 1 if the static posture was maintained for > 1 min or the motion was repeated > 4 times/min) and load scores (0: <2 kg; +1: 2–10 kg intermittent; +2: 2–10 kg static or repeated; +3: >10 kg or shock/rapid force build-up) were added to the respective posture scores. The RULA grand score (1–7) was then obtained by cross-referencing the A- and B-group final scores in RULA Table C. Risk levels were classified as follows: scores 1–2 = Level I (acceptable risk); 3–4 = Level II (low risk, further investigation may be needed); 5–6 = Level III (medium risk, further investigation and changes should be implemented soon); and 7 = Level IV (very high risk, immediate changes are required). A representative example of joint angle measurement using Kinovea software is shown in Fig. 1.

**Table 1:** Operational definitions of key posture moments for 12 operating room nursing procedures.

No.	Task	Definition of key posture moment	Rationale for selection
1	Surgical hand disinfection	While scrubbing the elbow: trunk slightly flexed forward, neck flexed to view the elbow, upper arm abducted 45°–90°, forearm flexed.	Highest load on shoulder and neck; static posture maintained.
2	Setting up and managing the sterile instrument table	When reaching for instruments on the far side of the table: trunk flexed forward ≥ 20°, one or both arms extended forward, neck flexed to view the table surface.	Maximum deviation of trunk and upper limbs from neutral position.
3	Patient positioning – lateral position	While lifting the patient’s near-side shoulder/hip and pushing toward the opposite side: trunk flexed forward with rotation, upper arm flexed 45°–90° and abducted, legs in a lunge stance.	Whole-body high-force coordination; high risk of lumbar torsion.
4	Patient positioning – prone position	At the moment when, after turning the patient, two nurses support the patient’s trunk (chest and iliac region) with their forearms and slowly lower them: trunk flexed forward, both arms extended forward in a lifting posture, legs in a lunge stance.	Whole-body force coordination; concentrated load on lumbar spine and shoulder joints; high requirement for eccentric muscle control.
5	Draping the surgical area	When unfolding and placing the large drape to the opposite side of the operative field: trunk flexed forward leaning over the operating table, upper arm abducted and extended forward (≥ 45°), neck flexed to view the surgical area.	Largest posture amplitude; concentrated load on upper limbs and trunk.
6	Needle threading (standing)	At the moment of passing the suture through the needle eye: neck deeply flexed to view the needle eye, upper arm slightly abducted and raised, forearm flexed, wrist finely rotating to adjust alignment.	Large static neck flexion angle; repetitive fine wrist rotation; high visual load.
7	Passing surgical instruments	When passing a bone hammer (approx. 2 kg) and striking it against the surgeon’s palm: upper arm slightly abducted and extended forward, forearm flexed, wrist rotated to complete the striking motion, neck fixed on the operative field.	Striking requires wrist rotation and force; significantly increased load on upper arm and forearm muscles; high-frequency repetitive task.
8	Post operative instrument counting and arranging	Bending down to count scattered instruments on the far side of the table: trunk flexed forward ≥ 20°, neck deeply flexed, arms extended forward.	Prolonged static forward flexion; significant static load on lumbar spine.
9	Post operative patient transfer	When moving an anesthetized patient from the operating table to the transfer stretcher: trunk flexed forward with rotation and exertion, upper arms extended forward to push/pull, legs in a lunge stance.	Whole-body high-force coordination; high-risk motion for low back injury.
10	Moving dressings	When bending from a low shelf to lift a 5 kg dressing pack: trunk deeply flexed forward, both arms holding the pack.	Highest lumbar load at the initiation of the lift.
11	Transporting a patient by stretcher	When pushing the stretcher rail to turn a corner: trunk inclined forward with lateral rotation and force, upper arms extended forward to push the handle, lower limbs in a forward-backward stance to bear weight.	During turning, inertia must be overcome and direction changed; forward flexion plus trunk rotation produce the highest load.
12	Moving equipment	At the moment of initiating movement of a laparoscopic system trolley (stacked with monitor, camera unit, light source, insufflator, etc.) from standstill: trunk inclined forward with exertion, upper arms extended forward to push the handle, lower limbs in a forward-backward stance to bear weight.	The laparoscopic system is heavy; maximum static friction must be overcome at initiation; whole-body force concentrated on low back and upper limbs.

## Quality control

**Fig. 2:**
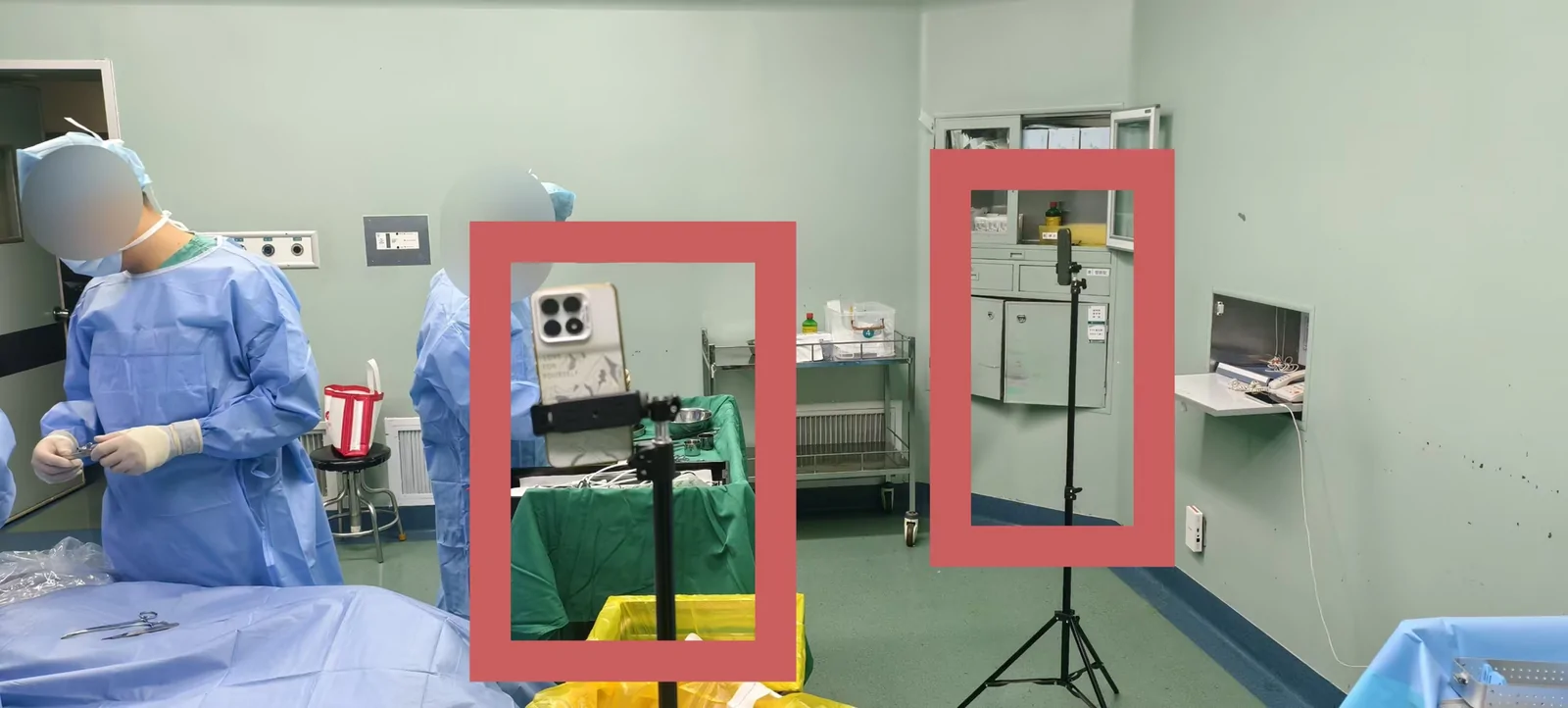
Video recording setup for ergonomic assessment. The figure shows the camera positioning for capturing representative posture frames Two smartphones on adjustable stands were placed at frontal and lateral views respectively to simultaneously record the complete procedure, enabling subsequent joint angle measurement using Kinovea software. The cameras were positioned to avoid interference with the surgical workflow and to protect patient privacy.

Prior to formal data collection, a pilot observation of all 12 procedures was conducted to refine the operational definitions of the key posture moments, ensuring clarity and reproducibility. All assessors received systematic training on the RULA scoring criteria and the PLIBEL checklist and participated in a pilot assessment to ensure rating consistency. For each procedure, 25 nurses were observed, and at least one complete operation cycle was video-recorded per nurse using the appropriate camera setup (Fig. 2 shows a representative example).The duration of observation varied by procedure type according to the nature of the task, with video recordings covering the entire operative cycle for each nurse. Typically, the recording duration for each procedure ranged from approximately 3 to 20 min depending on task complexity, with short-duration tasks such as needle threading requiring approximately 3–5 min and longer tasks such as patient positioning (lateral/prone) and postoperative patient transfer requiring approximately 10–20 min. The extracted key posture frames were independently reviewed by two assessors, and any disagreements in scoring were resolved through discussion and consensus.

### Statistical analysis

Statistical analyses were performed using SPSS version 26.0. Given the observational nature of this study and the deterministic scoring of PLIBEL and RULA, formal hypothesis testing was not conducted. Instead, descriptive statistics were used to summarize the ergonomic risk profiles across the 12 OR nursing procedures.

For RULA assessment, posture scores for each body region (upper arm, forearm, wrist, neck, trunk, and legs) and the grand score were reported as the median with minimum–maximum range across the 25 observations per procedure. The median was chosen to represent the typical risk level for each procedure, avoiding overestimation driven by rare extreme postures that do not reflect routine practice. The final RULA grand score (1–7) was used to classify each procedure into corresponding risk levels (Level I–IV) according to the standardized RULA protocol. For PLIBEL assessment, adverse ergonomic factors were identified as present when observed in more than 80% of observations, and the total number of identified factors was tabulated by procedure and body region.

Inter-rater reliability was assessed by comparing the independent scores assigned by the two assessors prior to consensus discussion. For RULA grand scores, the intraclass correlation coefficient (ICC) was calculated using a two-way random effects model with absolute agreement. For PLIBEL item-level assessments (dichotomous: hazard present/absent), Cohen‘s κ was calculated. All reliability analyses were performed using SPSS version 26.0.

## Results

### Inter-rater reliability

The inter-rater reliability between the two assessors was excellent for both assessment tools. For PLIBEL item-level assessments, Cohen‘s κ was 0.96, indicating almost perfect agreement. This exceptionally high agreement reflects the clear and straightforward nature of the PLIBEL criteria, which facilitated consistent judgments between the two assessors. For RULA grand scores, the ICC was 0.87 (95% CI: 0.74–0.94), indicating good to excellent agreement. These results demonstrate high consistency between the two assessors and support the reliability of the ergonomic risk assessments.

## Participant characteristics

A total of 25 operating room nurses participated in this study. The demographic and occupational characteristics of the participants are summarized in Table 2. The mean age was 34.5 ± 7.2 years (range: 24–47 years), and the mean body mass index (BMI) was 23.1 ± 1.6 kg/m² (range: 19.5–25.3 kg/m²). The majority of participants were female (*n* = 22, 88.0%). In terms of professional titles, 10 nurses (40.0%) held junior titles, 9 (36.0%) held intermediate titles, and 6 (24.0%) held senior titles. The mean duration of operating room experience was 9.8 ± 6.5 years (range: 1–24 years), with 12 nurses (48.0%) having less than 5 years, 6 (24.0%) having 5–10 years, and 7 (28.0%) having more than 10 years of OR experience.

**Table 2:** Demographic and occupational characteristics of participating operating room nurses (*n* = 25).

Characteristic	Value
Age (years), mean ± SD (range)	34.5 ± 7.2 (24–47)
Sex, n (%)	
Female	22 (88.0)
Male	3 (12.0)
Body mass index (kg/m²), mean ± SD (range)	23.1 ± 1.6 (19.5–25.3)
Professional title, n (%)	
Junior (nurse)	10 (40.0)
Intermediate (senior nurse)	9 (36.0)
Senior (supervisor nurse or above)	6 (24.0)
OR experience (years), mean ± SD (range)	9.8 ± 6.5 (1–24)
< 5 years, n (%)	12 (48.0)
5–10 years, n (%)	6 (24.0)
> 10 years, n (%)	7 (28.0)

### PLIBEL adverse ergonomic factor identification

A total of 243 adverse ergonomic factors were identified by PLIBEL across the 12 OR nursing procedures (Table 3). The number of identified factors per procedure ranged from 13 (surgical hand antisepsis) to 25, with lateral positioning, prone positioning, and postoperative patient transfer each accumulating 25 factors. All 12 procedures shared two common adverse factors: unsupported standing posture (Item 7a) and fatiguing leg work (Item 7c). The neck–shoulder–upper-back and elbow–forearm–hand regions accounted for the largest proportion of adverse factors across procedures. Among the five procedures subsequently rated Level IV by RULA, the lower back region consistently showed the co-occurrence of multiple adverse factors, including severe forward bending (9b), heavy load lifting (10), difficult grip (11), and confined work space (13).

### RULA ergonomic risk assessment

The RULA scores for the 12 procedures, reported as the median (minimum–maximum) across 25 observations per procedure, are presented in Table 4. In the typical posture tier, the RULA grand scores ranged from 4 to 7, corresponding to risk levels II (low risk) through IV (very high risk). Five procedures scored 7 (Level IV, very high risk): lateral positioning, prone positioning, postoperative patient transfer, stretcher patient transport, and equipment transport. Three procedures scored 6 (Level III, medium risk): surgical hand antisepsis, sterile instrument table preparation, and surgical draping. Three additional procedures scored 5 (Level III, medium risk): instrument transfer, postoperative instrument counting and sorting, and dressing transport. Needle threading scored 4 (Level II, low risk). A-group scores were highest for lateral positioning, prone positioning, and postoperative patient transfer (all = 7), reflecting large upper arm postural deviations and high external loads (≥ 10 kg). B-group scores were also highest for the same three procedures (all = 8), driven by severe trunk flexion, asymmetric leg loading, and high external loads.

**Table 3:** Identification of Adverse Ergonomic Factors Using PLIBEL Method in 12 Operating Room Nursing Procedures (*n* = 25).

Procedureno.		Nursing procedure	Neck/shoulder/upper back	Elbow/forearm/hand	Foot	Knee/Hip		Lower back	Totalfactors
1		Surgical hand antisepsis	1a, 1c, 2, 3	4a, 4c, 6b, 6c	7a, 7c	8b		9a, 9c	13
2		Sterile instrument table setup	1a, 1c, 2, 3	4a, 4b, 4c, 6a, 6c	7a, 7c	8b		9a, 9b, 9c, 9d, 10, 11, 12, 13	21
3		Patient positioning (lateral)	1a, 1b, 1c, 2, 3	4a, 4b, 4c, 4d, 5, 6a, 6b, 6c	7a, 7c	8b		9a, 9b, 9c, 9d, 10, 11, 12, 13	25
4		Patient positioning (prone)	1a, 1c, 2, 3	4a, 4b, 4c, 4d, 5, 6a, 6b, 6c	7a, 7c	8b		9a, 9b, 9c, 9d, 10, 11, 12, 13	25
5		Surgical draping	1a, 1c, 2, 3	4a, 4b, 4c, 4d, 6a, 6b, 6c	7a, 7c	8b		9a, 9b, 9c, 10, 11, 13	20
6		Needle threading (standing)	1a, 1c, 2, 3	4a, 4c, 4d, 6a, 6b, 6c	7a, 7c	8b		9a, 12	16
7		Surgical instrument passing	1a, 1c, 2, 3	4a, 4b, 4c, 4d, 6a, 6b, 6c	7a, 7c	8b		9a, 9c, 9d, 12	19
8		Postoperative instrument counting	1a, 1c, 2, 3	4a, 4b, 4c, 6a, 6b, 6c	7a, 7c	8b		9a, 9b, 9c, 10, 11, 12, 13	21
9		Postoperative patient transfer	1a, 1c, 2, 3	4a, 4b, 4c, 4d, 5, 6a, 6b, 6c	7a, 7c	8b		9a, 9b, 9c, 9d, 10, 11, 12, 13	25
10		Dressing material handling	1a, 1c, 3	4b, 4c, 5, 6b, 6c	7a, 7c	8a, 8b		9b, 9c, 9d, 10, 11, 13	20
11		Stretcher patient transport	1a, 1c, 3	4b, 4c, 5, 6b, 6c	7a, 7c	8b		9a, 9b, 9c, 9d, 10, 11, 13	19
12		Medical equipment transport	1a, 1c, 3	4a, 4b, 4c, 5, 6b, 6c	7a, 7c	8b		9a, 9b, 9c, 9d, 10, 11, 13	19

PLIBEL hazard factor codes: 1a = repeated or sustained neck flexion; 1b = repeated or sustained neck extension; 1c = repeated or sustained neck side-bending/rotation; 2 = unsuitable working height with shoulder elevation or unsupported arms; 3 = repeated or unsupported arm reach forward/sideways; 4a = repetitive or sustained hand lifting/manipulation; 4b = heavy hand load; 4c = difficult hand grip; 4d = sustained awkward hand position; 5 = repeated support of heavy loads or uncomfortable lifting/pushing/pulling; 6a = hand/forearm twisting work; 6b = forceful hand/forearm work; 6c = awkward hand/forearm posture; 7a = unsupported standing work without seat; 7b = fatiguing footwork; 7c = fatiguing leg work (prolonged standing or single-leg load bearing); 8a = prolonged kneeling or squatting; 8b = unsupported standing posture (knee/hip); 9a = mild back flexion; 9b = severe back flexion; 9c = back side-bending or mild rotation; 9d = severe back rotation; 10 = heavy lifting or carrying; 11 = difficult grip; 12 = high visual demand; 13 = restricted workspace or material access.

**Table 4:** RULA Assessment Results for 12 Operating Room Nursing Procedures (Median, *n* = 25).

No.	Procedure	Upper arm	Forearm	Wrist	Wrist twist	Muscle use	Load	Group A score	Neck	Trunk	Legs	Muscle use	Load	Group B score	Total score	Risk level
1	Surgical hand antisepsis	4	2	2	1	1	0	4	3	3	1	1	0	4	6	Level III
2	Sterile instrument table setup	4	2	3	1	1	0	4	3	3	1	1	0	4	6	Level III
3	Patient positioning (lateral)	5	2	4	1	1	3	7	3	4	2	1	3	8	7	Level IV
4	Patient positioning (prone)	5	2	4	1	1	3	7	3	4	2	1	3	8	7	Level IV
5	Surgical draping	4	2	3	1	1	0	4	3	3	1	1	0	4	6	Level III
6	Needle threading (standing)	3	2	2	1	1	0	3	3	2	1	1	0	3	4	Level II
7	Surgical instrument passing	3	2	2	1	1	1	4	3	2	1	1	1	3	5	Level III
8	Postoperative instrument counting	3	2	2	1	1	0	3	3	3	1	1	0	4	5	Level III
9	Postoperative patient transfer	5	2	4	1	1	3	7	3	4	2	1	3	8	7	Level IV
10	Dressing material handling	4	2	2	1	1	1	4	2	3	2	1	1	4	5	Level III
11	Stretcher patient transport	4	2	2	1	1	3	5	3	4	2	1	3	7	7	Level IV
12	Medical equipment transport	4	2	2	1	1	3	5	3	4	2	1	3	7	7	Level IV

RULA (Rapid Upper Limb Assessment) risk levels: Level I (score 1–2) = acceptable posture; Level II (score 3–4) = low risk, may need further investigation; Level III (score 5–6) = medium risk, changes may be needed; Level IV (score 7) = high risk, changes needed urgently. Group A (Upper Limb): includes upper arm, forearm, wrist, and wrist twist scores. Group B (Neck/Trunk/Legs): includes neck, trunk, and leg posture scores. Muscle Use and Load scores are added to group scores as appropriate.

## Discussion

This study employed PLIBEL and RULA in combination to systematically identify and quantify adverse ergonomic factors across 12 common OR nursing procedures. The PLIBEL analysis identified a total of 243 adverse factors, with all 12 procedures sharing unsupported standing posture (Item 7a) and fatiguing leg work (Item 7c) as common exposures. The RULA assessment, conducted on the typical posture tier, revealed grand scores ranging from 4 to 7, with five procedures—lateral positioning, prone positioning, postoperative patient transfer, stretcher transport, and equipment transport—reaching the maximum score of 7 (Level IV, very high risk). These findings converge to indicate that patient positioning and patient transfer tasks constitute the highest-priority targets for ergonomic intervention in the OR.

The present findings are consistent with the broader epidemiological literature on WMSDs among perioperative nurses. Clari et al., in a systematic review and meta-analysis encompassing 22 international studies and 3,590 perioperative nurses, reported pooled WMSDs prevalences of 62% for the lower back, 47% for the knees, and 44% for the shoulders^3^. Tavakkol et al. demonstrated that OR nurses exhibited the highest REBA (10/15) and RULA (7/7) scores among all hospital departments, with 98% reporting musculoskeletal pain in the preceding 12 months^4^. A global systematic review and meta-analysis further confirmed that operating room personnel have the highest prevalence of WMSDs among all healthcare workers, with the lower back being the most commonly affected region^13^. Our results extend these prevalence data by providing procedure-level resolution of ergonomic risk, using a dual-tool methodology that captures both the breadth of hazard exposure (via PLIBEL) and the severity of postural risk (via RULA).Specifically, our study offers three main contributions. First, we systematically applied this combined framework across 12 common OR nursing procedures, covering the entire workflow from preoperative preparation to postoperative finishing — a broader scope than previous studies that have largely used single tools or assessed limited task sets. Second, PLIBEL provides a comprehensive hazard inventory, while RULA delivers graded risk quantification, enabling a complete risk characterization from qualitative screening to quantitative grading. Third, based on our procedure-level risk stratification, we propose tiered intervention strategies tailored to different risk levels (Levels II–IV), providing actionable, priority-oriented guidance for clinical practice.

Among the 12 procedures examined, those involving patient positioning (lateral and prone) and patient transfer (postoperative bed transfer; stretcher transport) exhibited the highest risk profiles, accumulating the maximum PLIBEL factor count (19–25 factors) and a RULA score of 7. The mechanisms underlying these elevated risk scores, however, differed between the two categories. Positioning procedures involve sustained lifting and holding of anesthetized patients at a distance from the body, combining high external loads (> 10 kg) with substantial trunk flexion, lateral bending, and twisting—a constellation of risk factors that has been identified as particularly hazardous in longitudinal studies of WMSDs^1^. In our PLIBEL analysis, these procedures consistently demonstrated the co-occurrence of severe forward bending (Item 9b), heavy load lifting (Item 10), difficult grip (Item 11), and confined work space (Item 13) in the lower back region. In contrast, transfer procedures were characterized by short-duration, high-force exertions involving pushing, pulling, and twisting, with lower A-group scores (5 vs. 7) but comparably elevated B-group scores, suggesting that the risk in transfer tasks is concentrated in the trunk rather than the upper limbs. This distinction has practical implications for intervention design: positioning tasks may benefit most from mechanical lift assists that reduce the load moment arm, whereas transfer tasks may be better addressed through friction-reducing devices and team-based protocols. Previous studies have confirmed that patient handling devices, including ceiling lifts, air-assisted transfer devices, and slide sheets, significantly reduce biomechanical loads on the lower back and upper extremities during lateral patient transfers, with ceiling lifts and air-assisted devices producing the greatest reductions in hand force and spinal loading^14,15^.

Procedures scoring in the medium-risk range (RULA 5–6) accounted for six of the twelve operations. Although not reaching the very-high-risk threshold, sterile instrument table preparation and postoperative instrument counting each accumulated 21 PLIBEL-identified adverse factors, driven largely by sustained trunk flexion, repetitive hand movements, and prolonged standing. Needle threading, the sole Level II procedure (RULA = 4), nonetheless exhibited 16 PLIBEL factors, predominantly from sustained deep neck flexion, fine repetitive wrist rotation, and high visual demands. These findings align with evidence that cumulative, low-to-moderate intensity exposures over extended periods contribute substantially to WMSDs development among healthcare workers. Jacquier-Bret and Gorce, in a systematic review of body area WMSDs among healthcare professionals, reported that maintenance and repetition of awkward postures were the most frequently cited causes across all professional groups^2^.

A key contribution of this study lies in demonstrating the complementary value of PLIBEL and RULA when used in tandem. PLIBEL, as a qualitative screening tool, provided a broad inventory of adverse factors (totaling 243 across 12 procedures). This broad scanning capability captured risks that RULA alone would not fully reflect—notably, unsupported standing posture (Items 7a/7c), which was universally present yet receives limited weighting in the RULA framework. RULA, in turn, converted qualitative observations into quantifiable risk grades, enabling direct inter-procedure comparison and a clear intervention prioritization. In several procedures, PLIBEL identified numerous adverse factors while RULA scores remained moderate (e.g., sterile instrument table preparation: 21 factors, RULA = 6), suggesting that the postural load had not reached the extreme, but the cumulative ergonomic burden was substantial. Conversely, for positioning and transfer procedures, both tools converged on the highest risk, confirming that these tasks warrant immediate ergonomic attention. This complementary assessment paradigm—combining broad hazard screening with graded risk quantification—has been advocated for the comprehensive evaluation of WMSDs risk across diverse occupational settings^14,16^. Our study extends this approach to the OR nursing context, demonstrating that the PLIBEL–RULA combination can generate actionable, stage-specific risk profiles that are directly translatable to clinical practice.

From a practical standpoint, these findings suggest a multi-level approach to ergonomic improvement in the OR. First, for the Level IV procedures identified in this study (patient positioning, patient transfer, and equipment transport), specific ergonomic measures may be considered. For positioning tasks, mechanical lifting aids (e.g., ceiling lifts, air-assisted transfer devices, or slide sheets) should be used to reduce manual load, with adequate staff assistance (at least 2–3 trained personnel) to distribute physical demands, and the operating table should be adjusted to an appropriate height before positioning to minimise trunk flexion. For transfer tasks, friction-reducing devices (e.g., sliding boards or roller sheets) should be employed to facilitate horizontal transfers, height-adjustable stretchers should be used to minimise lifting distance, and team-based protocols with clear role assignment should be implemented to reduce unnecessary individual exertion. For equipment transport, heavy equipment trolleys should be fitted with large-diameter wheels and regularly maintained to minimise rolling resistance, and motorised push-assist devices should be used where feasible for laparoscopic towers and other heavy equipment, alongside training on proper pushing/pulling techniques. A systematic review has confirmed that patient transfer assistive devices, notably ceiling lifts and air-assisted devices, are effective in reducing WMSDs risk among nurses, with some studies reporting up to a 59.8% reduction in WMSDs incidence and a 90% reduction in missed workdays^15^. Second, for the universally present factors of prolonged standing (Items 7a/7c), anti-fatigue floor mats and height-adjustable workstations should be introduced to mitigate lower-limb static loading. Third, work process restructuring, including the scheduling of microbreaks during prolonged procedures and the rotation of high-load tasks among team members, should be incorporated into standard OR protocols. A multicenter longitudinal cohort study demonstrated that structured microbreak interventions (2–3 min breaks every 30 min during procedures lasting > 60 min) produced sustained improvements in MSDs outcomes, with a 20.9% point greater reduction in MSDs prevalence compared to controls, alongside significant improvements in psychological wellbeing and patient safety indicators^17^. A systematic review of ergonomics training and intra-operative microbreaks further supports the integration of both strategies to reduce musculoskeletal injuries among OR personnel^18^. Furthermore, multicomponent participatory ergonomic interventions that combine physical activities, exercise, educational programs, and patient handling devices have been shown to be more effective in reducing WMSDs than single-component approaches^19^. It should be noted that these suggested interventions were not evaluated in the present study, and their effectiveness in this specific context requires confirmation through future interventional research.

Several limitations of this study should be acknowledged. First, while RULA scoring was based on objective joint angle measurements using Kinovea software, PLIBEL identification relied on observers’ judgment of the entire procedural workflow, which may introduce subjective variability. This limitation was mitigated through dual-assessor evaluation and consensus resolution. Second, this study was designed as an ergonomic exposure assessment and did not include clinical outcome measurements, such as musculoskeletal pain scores or WMSD prevalence among the participating nurses. Consequently, we were unable to establish direct exposure–effect relationships between the identified ergonomic risk profiles and actual musculoskeletal health status. Future longitudinal studies should combine objective ergonomic assessments with validated clinical outcome tools (e.g., the Nordic Musculoskeletal Questionnaire) to verify these associations. Third, this study was conducted at a single tertiary hospital with 25 participating nurses. This single-center design was chosen to ensure consistency in equipment, environmental conditions, and workflow across all observations, thereby minimizing confounding variables and enhancing internal validity. However, this necessarily limits the generalizability of our findings to other OR settings with different equipment configurations, staffing models, or surgical volumes. The sample size of 25 nurses was determined based on sample size requirements for reliability estimation (see Methods), and was not powered for formal statistical comparisons between procedures or for generating generalizable population estimates. Therefore, the findings should be interpreted as preliminary ergonomic profiling rather than representative estimates for all OR nurses. Fourth, the study employed a cross-sectional design that captured ergonomic risk at defined key posture moments; real-time dynamic variations in posture throughout prolonged procedures were not continuously monitored. Consequently, the single-frame assessment approach may underestimate cumulative exposure and fail to capture the full range of postural variability that occurs during routine OR nursing tasks—a important limitation given that both factors are recognized as important determinants of WMSD risk. Future studies employing continuous motion capture technologies (e.g., inertial measurement units) could better characterize the full spectrum of postural variability and cumulative exposure, thereby providing a more comprehensive assessment of ergonomic risk. In addition, future intervention studies are warranted to evaluate whether ergonomic training programmes or the introduction of assistive devices (e.g., mechanical lifts, friction-reducing transfer aids, or height-adjustable equipment) can effectively reduce PLIBEL and RULA scores in real-world OR settings, thereby translating the risk profiles identified in the present study into actionable preventive strategies.

## Data Availability

The datasets generated and analyzed during the current study are available from the corresponding author on reasonable request.
